# Combined use of interferon alpha-1b, interleukin-2, and thalidomide to reverse the AML1-ETO fusion gene in acute myeloid leukemia

**DOI:** 10.1007/s00277-021-04621-w

**Published:** 2021-07-27

**Authors:** Ruihua Mi, Lin Chen, Haiping Yang, Yan Zhang, Jia Liu, Qingsong Yin, Xudong Wei

**Affiliations:** 1 The Affiliated Cancer Hospital of Zhengzhou University/Henan Cancer Hospital, Zhengzhou, 450008 China; 2 The First Affiliated Hospital, and College of Clinical Medicine of Henan University of Science and Technology, Luoyang, 471003 China; 3 Anyang District Hospital, Anyang, 455000 China

**Keywords:** Myeloid leukemia, Interferon, Interleukin-2, Thalidomide, AML1-ETO fusion gene

## Abstract

This study aims to explore the effect of the ITI (interferon alpha-1b, thalidomide, and interleukin-2) regimen on the AML1-ETO fusion gene in patients with t(8;21) acute myeloid leukemia (AML) who were in hematologic remission but positive for the AML1-ETO fusion gene. From September 2014 to November 2020; 20 patients with AML (15 from The Affiliated Cancer Hospital of Zhengzhou University, 4 from The First Affiliated Hospital; and College of Clinical Medicine of Henan University of Science and Technology, and 1 from Anyang District Hospital) with hematological remission but AML1-ETO fusion gene positivity were treated with different doses of the ITI regimen to monitor changes in AML1-ETO fusion gene levels. Twenty patients were treated with a routine dose of the ITI regimen, including 13 males and 7 females. The median patient age was 38 (14–70 years). The fusion gene was negative in 10 patients after 1 (0.5 ~ 8.6) month, significantly decreased in 4 patients after 2.8 (1 ~ 6) months, increased in 4 patients, and unchanged in 2 patients. The 4 patients with elevated levels of the fusion gene were treated with an increased dose of the ITI regimen, and all four patients became negative, for a total effective rate of 90%. The ITI regimen reduces AML1-ETO fusion gene levels in patients with AML who are in hematologic remission but are fusion gene**–**positive. Improvement was observed in patients’ response to a higher dose administration, and patients tolerated the treatment well.

At present, acute myeloid leukemia (AML) prognosis stratification is primarily performed according to cytogenetics and molecular biology [1], but with the improvement of detection methods, minimal residual disease (MRD) has also become an independent prognostic factor for AML [2]. The lower the level of MRD, the more patients may have the advantage of long-term survival. The prognosis of patients with AML with t(8;21) chromosome abnormality, known as the AML1-ETO fusion gene, is relatively good, but clinically, the prognosis of these patients is heterogeneous: some patients cannot achieve AML1-ETO fusion gene negativity, or the fusion gene becomes positive again after becoming negative during treatment, conveying a potentially poor prognosis. Hematopoietic stem cell transplantation may be the best treatment for these patients but for those patients who cannot be transplanted. There is no consensus on how to perform follow-up intervention treatment. Interferon α-1b (IFN-α-1b) and thalidomide (Tha) combined with interleukin (IL)-2 (the ITI regimen) were used to treat 20 patients with AML whose AML1-ETO fusion gene was persistently positive or had turned positive again after becoming negative. The results are reported as follows.

## Materials and methods

### Cases

This study included 20 patients with AML with AML1-ETO fusion gene positivity from September 2014 to November 2020, including 15 from The Affiliated Cancer Hospital of Zhengzhou University; 4 from The First Affiliated Hospital, and College of Clinical Medicine of Henan University of Science and Technology; and 1 from Anyang District Hospital. All patients were informed about the study and provided consent in writing. All patients were diagnosed by routine blood tests, bone marrow morphology, flow immunotyping, cytogenetics, and molecular biology. There were 13 males and 7 females with a median age of 38 (14–70 years). According to the guidelines for the diagnosis and treatment of Chinese adult AML [3], prognosis was classified into low-risk (*n* = 13), medium-risk (*n* = 6), and high-risk (*n* = 1) groups. Patients with FLT3-ITD mutations were treated with sorafenib before induction and consolidation. Among the 20 patients, 18 achieved morphological remission after one course of treatment, and 2 patients achieved morphological remission after 2 courses, 9 of which were persistently positive for the fusion gene. After consolidation therapy, the fusion gene changed from negative to positive in 3 cases. During consolidation therapy, the fusion gene did not maintain stable negativity in 8 cases. Detailed clinical data of the 20 patients are shown in Tables 1 and 2.

**Table 1:** Initial clinical data of 20 AML patients who received intervention therapy with the ITI regimen

Patient no	Sex	Age (year)	Chromosome karyotype	Gene mutation	WBC (× 109/L)	NCCN prognosis stratification
1	Male	68	46,XY,t(8;21)(q22;q22)[20]	FLT3-ITD( +)	46.12	High risk
2	Male	70	45,X,-Y,t(8;21) (q22;q22)[20]	c-KIT( +)	28.00	Medium risk
3	Male	32	46,XY, t(8;21)(q22;q22) [17]/46.XY[3]	TYK2, CROCC, PTPRT, MUC16( +)	53.30	Low risk
4	Male	32	46,XY,t(8;21)(q22;q22)[10]	(-)	46.80	Low risk
5	Male	29	45,X,-Y,t(8;21) (q22;q22)[4]/46,idem, + 19[5]/46,XY[1]	c-KIT( +)	12.79	Medium risk
6	Male	37	46,XY,t(8;21)(q22;q22)[10]	TET2(SNP), IKZF1( +)	9.67	Low risk
7	Female	42	45,X, X,t(8;21) (q22;q22)[7]	c-KIT( +)	27.50	Low risk
8	Male	24	46,XY,t(8;21)(q22;q22)[10]	(-)	4.63	Low risk
9	Male	31	46,XY,t(8;21)(q22;q22)[10]	(-)	1.93	Low risk
10	Female	59	46,XX,t(8;21)(q22;q22)[5]	c-KIT( +)	215	Medium risk
11	Female	61	45,X,X,t(8;21) (q22;q22)[3]/46,XX[1]	N-RAS, c-Kit, SMCIA( +)	9.9	Medium risk
12	Male	14	46,XY,t(8;21) (q22;q22) [20]	(-)	5.19	Low risk
13	Female	39	45,X, X,t(8;21) (q22;q22)[20]	(-)	43.27	Low risk
14	Male	47	46,XY,t(8;21)(q22;q22)[5]	DNMT3A( +)	3.90	Low risk
15	Female	36	46,XX,t(8;21)(q22;q22)[15]	TET2( +)	5.23	Low risk
16	Male	57	46,XY,t(8;21)(q22;q22)[20]	ABCB1( +)	3.27	Low risk
17	Male	35	49,XY, + 4, + 4, + 8,t(8;21)(q22;q22)[5]	(-)	4.65	Low risk
18	Male	26	46,XY,t(8;21)(q22;q22)[4]/46,XX[2]	C-Kit( +)	6.79	Medium risk
19	Female	59	46,XX[1]/46,XX,t(8;21) (q22;q22) [7]	C-Kit( +)	10.63	Medium risk
20	Male	57	46,XY,t(8;21)(q22;q22)[20]	(-)	7.26	Low risk

**Table 2:** Pretreatment experience of 20 patients with AML treated with the ITI regimen and the changes in gene levels before and after treatment

Patient no	Pretreatment experience	Positive nature of fusion gene	Gene level of pretreatment by RCR	MRD level of pretreatment by RCR flow cytometry	Bone marrow morphology before treatment	Conventional dose application time(m)	Gene level of after-treatment	Follow-up processing and “ITI” treatment cycles
1	IA, IA, ID-Ara-C, ID-Ara-C, CAG	Turn negative and then turn positive again in the course of chemotherapy	0.11%	(-)	Active obviously, no abnormal neutrophils were found	1	Negative	Continue routine dose application; 6
2	Venetoclax + AZA, IA, ID-Ara-C, DCAG, PD-1 monoclonal antibody, PD-1 monoclonal antibody + imatinib × 3	Turn negative and then turn positive again in the course of chemotherapy	0.21%	(-)	Active, no abnormal neutrophils were found	0.5	Negative	Continue routine dose application; 4
3	DA, HD-Ara-C, HD-Ara-C, ID-Ara-C + IDA, HA + dasatinib	Continue not to turn negative	0.03%	(-)	Active, 0.5% primordial granulocyte	8.6	Negative	Continue routine dose application; 13
4	DHA, EA, HD-Ara-C, HD-Ara-C, HD-Ara-C	Turn negative and then turn positive again in the course of chemotherapy	1.39%	0.14%	Active, no abnormal neutrophils were found	3.6	0.13%	allo-HSCT; 3.6
5	IA, HD-Ara-C, CAG, allo-HSCT	Turn negative and then turn positive again in the course of chemotherapy	1.05%	0.09%	Active, 0.2% primordial granulocyte	2	Relapse	Give up treatment; 2
6	IA, HD-Ara-C, dasatinib + CAG, CHAG, MA, DCHAG	Continue not to turn negative	0.4%	(-)	Active, 0.5% primordial granulocyte	1.5	Negative	Continue routine dose application; 36
7	IA, imatinib + CAG, HD-Ara-C + imatinib, HA + imatinib	Turn positive 16 months after the end of the last chemotherapy	0.02%	(-)	Active, no abnormal neutrophils were found	1	0.04%	The gene turned negative after 5.5 months of adding application; 36
8	IA, HD-Ara-Cx2, HA	Continue not to turn negative	0.35%	(-)	Active, 0.2% primordial granulocyte	1	Negative	allo-HSCT; 1
9	IA, HD-Ara-Cx2, DCAG, DCHAG	Continue not to turn negative	0.13%	(-)	Active, no abnormal neutrophils were found	1	Negative	Continue routine dose application; 30
10	IA, ID-Ara-Cx2, dasatinib + IA, CHG	Turn negative and then turn positive again in the course of chemotherapy	0.24%	(-)	Active, no abnormal neutrophils were found	1	Negative	Continue routine dose application; 6
11	DA, DA, ID-Ara-C, ID-Ara-C, EA, HA, DA, ID-Ara-C, DA	Continue not to turn negative	0.11%	(-)	Active, no abnormal neutrophils were found	6	0.03%	Continue routine dose application; 8
12	IA, HD-Ara-Cx2, HA, DCAG, DCHAG	Turn negative and then turn positive again in the course of chemotherapy	0.17%	(-)	Active, no abnormal neutrophils were found	2	Negative	Continue routine dose application; 5
13	HAA, HD-Ara-Cx3, DA, AA	Turn negative and then turn positive again in the course of chemotherapy	0.16%	(-)	Active, no abnormal neutrophils were found	1	0.22%	The gene turned negative after 3.5 months of adding application; 7
14	IA, IA, HD-Ara-Cx2, HA, DCAG	Continue not to turn negative	0.21%	(-)	Active, no abnormal neutrophils were found	1.5	Relapse	allo-HSCT; 1.5
15	IA, HD-Ara-Cx3, CAG, DCHAG	Continue not to turn negative	0.13%	(-)	Active, no abnormal neutrophils were found	1	Negative	Continue routine dose application; 9
16	DCAG, IA, HD-Ara-Cx3	Turn positive 6 months after the end of the last chemotherapy	0.03%	(-)	Active, no abnormal neutrophils were found	2	Negative	Continue routine dose application; 25
17	IA, IA, HD-Ara-Cx3, DA	Turn negative and then turn positive again in the course of chemotherapy	0.08%	(-)	Active, no abnormal neutrophils were found	1	0.05%	allo-HSCT; 1
18	IA, HD-Ara-Cx3, HA, imatinib + DCAG	Continue not to turn negative	0.14%	(-)	Active, no abnormal neutrophils were found	2	0.03%	allo-HSCT; 2
19	DA, DA, ID-Ara-Cx2, imatinib + DCAG, imatinib + DCHAG	Continue not to turn negative	0.09%	(-)	Active, no abnormal neutrophils were found	1	0.16%	The gene turned negative after 4 months of adding application; 8
20	IA, HD-Ara-Cx3, IA, DA	Turn positive 8 months after the end of the last chemotherapy	0.25%	(-)	Active, no abnormal neutrophils were found	1	0.28%	The gene turned negative after 3 months of adding application; 10

*IA*, IDA + Ara-C; *ID-Ara-C*, intermediate dose Ara-C; *CAG*, Acla + Ara-C + G-CSF; *DCAG*, DEC + CAG; *DA*, DNR + Ara-C; *HD-Ara-C*, high-dose Ara-C; *HA*, HHT + Ara-C; *DHA*, HHT + DNR + Ara-C; *EA*, VP-16 + Ara-c; *allo-HSCT*, allogeneic hematopoietic stem cell transplantation; *CHAG*, HHT + CAG; *DCHAG*, DEC + CHAG; *MA*, MITO + Ara-C; *DCHAG*, DCAG + HHT; *HAA*, HA + Acla; *AA*, Acla + Ara-C

### Chromosome karyotype analysis

Approximately 5–6 ml bone marrow fluid in heparin anticoagulant was obtained from patients’ bone marrow. After counting, cells were inoculated into a 15 ml medium according to a cell concentration of (1–2) × 10^6^/ml. Chromosomes were prepared according to a routine method. Cells were harvested after addition of hypotonic solution and were fixed, and R-banding and Giemsa staining were performed for karyotype analysis. Abnormal karyotypes are described according to the International Nomenclature System of Human Cell Genomics (ISCN2016). The remaining cell suspension was stored at − 20 ℃.

### Nucleic acid extraction from patients with AML

Bone marrow fluid (2–3 ml) was collected from patients’ bone marrow. DNA was extracted using the blood genomic DNA extraction kit from Tiangen Biochemical Technology (Beijing) Co., Ltd. RNA was extracted using TRIzol and was quantified using a NanoDrop 2000 microspectrophometer (Thermo Scientific, USA) for follow-up detection.

### Detection of gene mutation by AML high throughput sequencing

Hot spots of the target genes were amplified by PCR primers and sequenced using the Ion Torrent PGM sequencing platform (Thermo Fisher, USA). The sequenced data were analyzed by using the human genome database (HG19), COSMIC, 1000 Genomes, and dbSNP. The average gene coverage was greater than 99%, the average sequencing depth was 1500 × , the sequencing depth of the target area was greater than 1000 × , and the detection sensitivity was 5%.

### Detection of the AML1-ETO fusion gene by real-time fluorescence quantitative PCR

Bone marrow mononuclear cells were isolated according to a routine method, and total RNA was extracted using the OMEGA Micro Elute total RNA extraction reagent. The purity and concentration of RNA were determined by spectrophotometry. An absorbance A260/A280 ≥ 1.8 was taken as a qualifying sample. The obtained RNA was reverse transcribed by a reverse transcription kit to obtain a template DNA chain. cDNA was used as a template, and a PCR kit was used to complete PCR. Q-PCR: it was carried out on an ABI Prism 7500 Real-Time Fluorescence Quantitative PCR instrument (produced by ABI company in the USA). The reaction system is 10 μl, including upstream and downstream primers 0.1 μmol/l, TaqMan probe 0.2 mol/l, 2 × TaqManuniversal PCR Mastermix (ABI company) 5 μl, and cDNA 1 μl. PCR conditions were 50 ℃, 2 min, 95 ℃, 10 min, 95℃, 15 s, 62 ℃, 1 min, and 40 cycles. Abl was selected as the internal reference gene. AML1-ETO primers and probe sequences were designed by using the Primer express 2.0 software (produced by American ABI company). Upstream primer: 5′-CACCTACCACAGAGCCATCAAA-3′, downstream primer: 5′-ATCCACAGGTGAGTCTG GCATT-3′, probe: 5′–FAM-AACCTCGAAATCGTACTGAGAAGCACTCCA-TAMRA-3′. The expression level of AML1-ETO is calculated by the following formula: AML-ETO level (%) = AML-ETO copy number/abl copy number × 100% (coefficient of variation ≤ 5%).

### Detection of MRD levels by 10-color flow cytometry(FCM)

Three milliliters of bone marrow fluid in heparin anticoagulant were collected from the patients’ bone marrow and detected by FCM within 2 h, which was performed with a Navios (Beckman Coulter company, USA). Immunophenotypic data were collected in two tubes, and the number of cells obtained by FCM was more than 500,000. Antibody markers of myeloid leukemia were as follows: CD34-A700/CD117-APC/CD13-PE/CD38-A750/CD15-FITC/CD33-PC5.5/HLA-DR-Blu/CD45-ko(tube1) and CD34-A700/CD117-APC/CD7-PE/CD56-ECD/CD64-FTIC/CD19-CY5.5/CD45-ko (tube 2). The ratio of leukemic cells to the total number of nucleated cells in the bone marrow was taken as the MRD value (MRD ≥ 0.01% was considered positive, and MRD < 0.01% was considered negative).

### Treatment plan and treatment cycle

Routine therapeutic dosing of the ITI regimen was subcutaneous injection of 60 μg IFN-α-1b (product of Shenzhen Kexing Biological Engineering Co., Ltd.), subcutaneous injection of 1 million U IL-2 (Beijing Sihuan Biopharmaceutical Co., Ltd.), and 100 mg oral Tha (Changzhou Pharmaceutical Co., Ltd.) before bed every night. Every 28 days is a cycle. If a patient’s PLT was > 50 × 10^9^/L, 2 tablets of the oral compound Danshen (Guangdong Baiyunshan Hutchison Whampoa Traditional Chinese Medicine Co., Ltd, composition: *Salvia miltiorrhiza*, *Panax notoginseng*, borneol) were administered 3 times a day to promote blood circulation and prevent deep venous thrombosis. Ibuprofen was taken orally 1 h before IFN-α-1b to prevent fever. AML patients with positive FLT3-ITD mutations were given 400 mg sorafenib twice a day. The interval between the application of the “ITI” regimen and the last chemotherapy was 30 (25–35) days.

The treatment efficacy was evaluated after 1 cycle. If effective, rhIFN α-1b and IL-2 were used three times a week in the first year, rhIFN α-1b and IL-2 were reduced to twice a week in the second year, and rhIFN α-1b and IL-2 were reduced to once a week in the third year. Thalidomide was used continuously and stopped after 3 years. After the end of “ITI” regimen, patients with AML with positive FLT3-ITD mutation continued to take sorafinib and stopped using sorafinib for 2 years. If the curative effect was ineffective after 1 cycle, a drug can be added, and the curative effect can be evaluated again after 1 cycle. If it was ineffective, the treatment plan can be changed.

Changes involved increasing the ITI regimen therapeutic dose as follows: 200 mg/day thalidomide, 60 μg/day rhIFN α-1b once a day, and 1 million U/day IL-2 once a day with the rest as above.

### Safety assessment

The safety of each cycle was evaluated before and after treatment. According to the evaluation criteria of adverse reactions established by the WHO, adverse reactions were divided into grades 0 ~ IV. The primary adverse reactions included fever, chills, rash, and muscle pain.

### Follow-up

Follow-up was conducted by telephone or medical records. Twenty patients were followed up to November 30, 2020, and 1 patient was lost to follow-up. Recurrence refers to a proportion of bone marrow blast cells greater than > 5%, peripheral blood blast cells, or extramedullary infiltration after complete remission (CR). Relapse-free survival (RFS) refers to the time from the date of the application of the “ITI” regimen to the date of death due or any cause, recurrence, or last follow-up. Overall survival (OS) time was from the date of the application of the “ITI” regimen to the end of follow-up or the death of the patient.

### Statistical analysis

The SPSS 22.0 software was used for statistical analysis. The measurement data are expressed as the median (range), the rank sum test was used for comparison, and the chi-square test was used to compare the continuous data between groups. The log-rank test was performed to compare OS and PFS using the Kaplan–Meier method, and a survival curve was drawn. Differences were considered statistically significant *P* < 0.05.

## Results

### Curative effect evaluation

Among the 20 patients, 14 patients’ AML1-ETO fusion genes became negative, 4 patients’ fusion gene levels decreased significantly, and 2 patients’ levels were unchanged with a total effective rate of 90%.

### The effective rate of the conventional ITI regimen dose is 70%

Twenty patients were treated with a routine ITI regimen dose. The fusion gene of 10 patients became negative, and the median application time was 1 (range, 0.5–8.6 cycles). The median gene level before treatment was 0.15% (0.03–0.4%). No leukemia cells were found in the bone marrow, and MRD was negative by 10-color flow cytometry.

The fusion gene was significantly decreased in 4 patients, and the median application time was 2.8 (range, 1–6 cycles). Median gene levels were 0.08%, 0.11%, 0.14%, and 1.39% in pretreatment, respectively. No leukemia cells were found in the bone marrow, but MRD levels were negative, negative, negative, and 0.14% by 10-color flow cytometry. Gene levels after treatment were 0.05%, 0.03%, 0.03%, and 0.13%, and 3 patients of which were followed up with allo-HSCT.

Gene levels in 4 patients increased, and they were subsequently given additional treatment.

Two patients’ gene levels remained unchanged. One patient did not experience gene negativity during the previous treatment and then underwent allogeneic hematopoietic stem cell transplantation at which point the gene still did not turn negative. The patient experienced morphological recurrence after 2 cycles of treatment. In the other case, the gene did not become continuously negative during treatment, and in the process of transplantation preparation, morphological recurrence occurred when ITI was used for 1.5 cycles and was followed by allo-HSCT.

### The effect of the increased ITI regimen may be superior

Four patients with elevated gene levels after routine dose treatment were treated with an increased dose of the ITI regimen, and their pretreatment gene levels were 0.02%, 0.16%, 0.09%, and 0.25%. After one cycle of conventional dose treatment, their gene levels increased to 0.04%, 0.22%, 0.16%, and 0.28%, subsequently becoming negative at 5.5, 3.5, 4, and 3 cycles, respectively.

### Adverse reactions

The ITI regimen was well-tolerated as a whole, and the primary adverse reactions included fever (90%), chills (70%), and muscle pain (30%). All of the above reactions occurred during the initial use of the regimen and gradually resolved after a few days. One patient developed a mild skin rash that improved after antiallergic treatment. Numbness of the end of the hand and foot (15%) occurred in the increased dose group, 2 patients were treated with nutritional nerve therapy, and 1 patient had mild symptoms without special treatment.

Primary adverse reactions of the hematological system included mild myelosuppression and low incidence of infection. No obvious infection of the lung, gastrointestinal tract, skin/soft tissue, or death due to treatment-related complications occurred during the entire treatment, except for 1 patient who had obvious myelosuppression after receiving allo-HSCT.

### Survival analysis

**Fig. 1:**
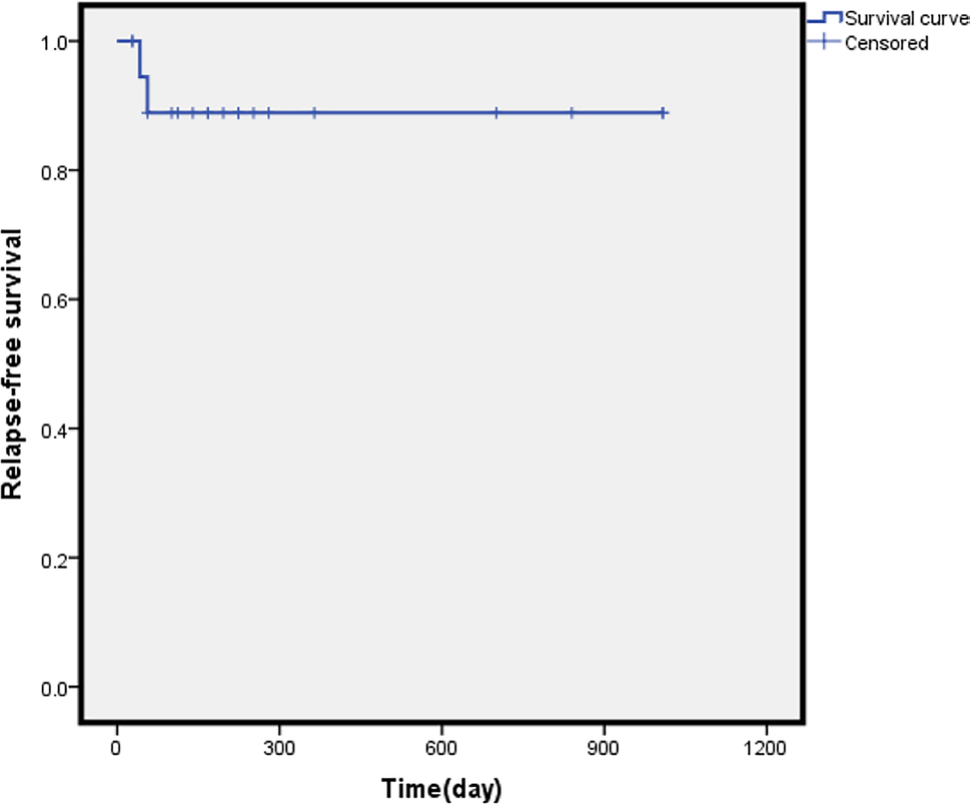
Relapse-free survival of 20 patients

**Fig. 2:**
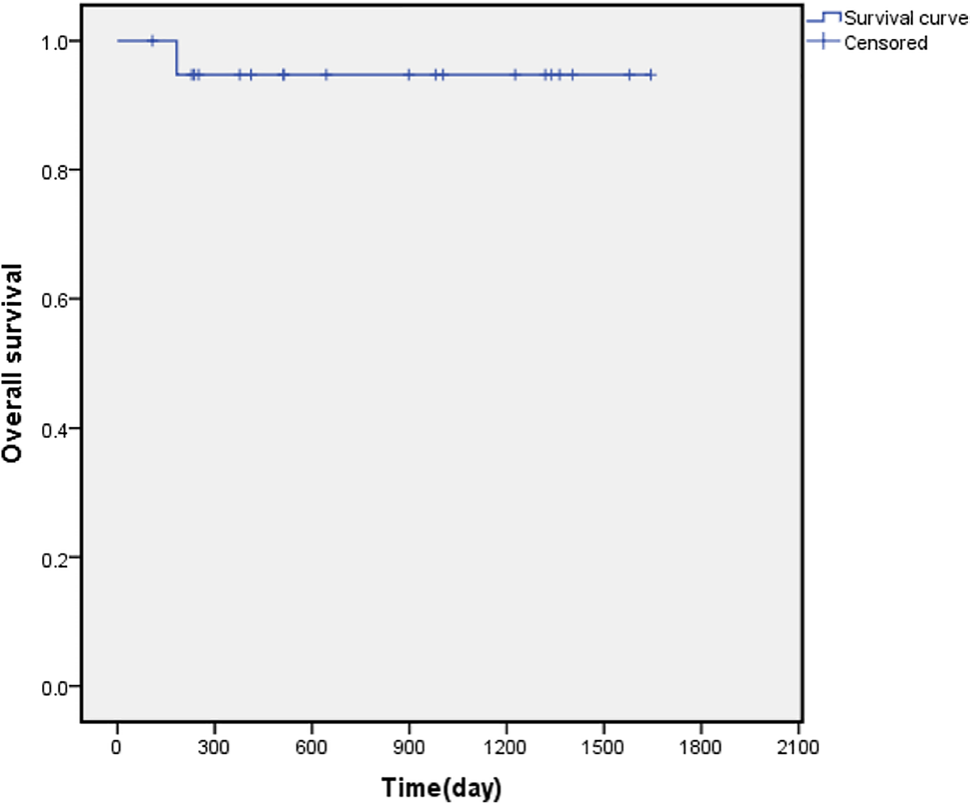
Overall survival of 20 patients

**Fig. 3:**
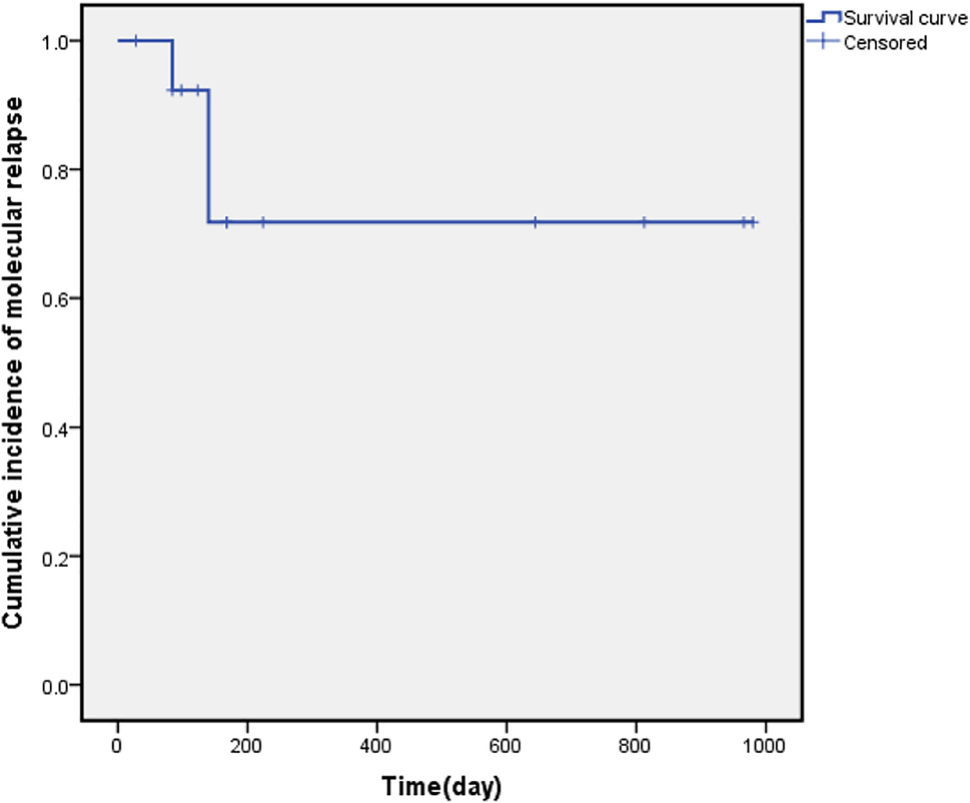
The cumulative incidence of molecular relapse in patients who achieved MRD negativity (from the time of MRD negativity achieved after the start of the IFI regimen)

During the median follow-up of 182 (28–1008 days), the median RFS and median OS of 20 patients were not reached, and the 2-year RFS and OS were 86.4% and 94.7%, respectively. Recurrence was noted in 2 cases (10%), and 1 patient died of disease recurrence and progression, as shown in Figs. 1 and 2. Fourteen cases of MRD turned negative after the application of the “ITI” regimen, of which 3 cases of MRD turned positive again, as shown in Fig. 3.

## Discussion

AML is a hematological tumor originating from hematopoietic stem cells. At present, chemotherapy is still the first-line treatment for AML. Allogeneic hematopoietic stem cell transplantation (allo-HSCT) is recommended for patients with medium-/high-risk prognosis [3]. Even if patients with AML achieve hematological remission in response to induction therapy, more than half of patients will relapse [4, 5]. In the traditional sense, recurrence refers to the proportion of blast cells in bone marrow or peripheral blood ≥ 0.05, but the number of cells examined by morphological examination is relatively small (200,500) and is greatly affected by bone marrow puncture and smear quality, which is related to the experience of the assessor [6].

MRD refers to a small number of leukemic cells that remain morphologically undetectable in patients with leukemia after morphological remission by induction chemotherapy or bone marrow transplantation. The aim of MRD detection is to identify leukemic cells that can cause recurrence with high sensitivity and specificity. At present, the primary detection methods of MRD are PCR and multiparameter flow cytometry (MFC), as well as the development of next-generation sequencing (NGS) and digital PCR. The sensitivity of MFC and PCR is different. MFC can reach a detection level of 10^−4^, PCR detection of a specific fusion gene can reach a 10^−6^ detection level, and NGS can reach a 10^−6^ detection level. Compared to PCR, the advantage of MFC is that it is suitable for more patients with AML, and the disadvantage is that MFC depends on the technology and experience of examiners. A total of 20 patients were enrolled in this study, AML1-ETO fusion gene levels were detected by PCR, and only 2 patients were MRD-positive in MFC.

Studies have confirmed that MRD targeting the AML1-ETO fusion gene can be used as a prognostic indicator [2]. However, it has been reported [7, 8]. that the optimal MRD threshold of PCR detection of this gene is related to different sampling sites and different treatment periods. Furthermore, the definition of the MFC MRD threshold remains controversial. ELN guidelines [9] believe that 0.1% has a good correlation with prognosis and is suitable to be used as the dividing point between negative and positive MRD. However, the clinical significance of less than 0.1% MRD still needs further study. The MRD threshold of patients in this study was set at 0.01%.

MRD can predict the risk of recurrence earlier [10]. Positive MRD after treatment indicates a high risk of recurrence, which is associated with shorter RFS and OS [2, 11–13].

The more direct purpose of testing MRD is to guide treatment intervention to provide patients with improved survival. A previous randomized controlled study showed that GO McAb significantly reduced MRD levels in patients with AMLt(8;21) [14]. According to the guidance of MRD, risk stratification of patients with AML with t(8;21) is performed again. High-risk patients are recommended to receive routine chemotherapy or autologous hematopoietic stem cell transplantation in those with a low risk of allo-HSCT. Studies have shown that reselection of treatment according to MRD risk stratification improves disease-free survival and overall survival in these patients [12].

Patients with MRD positivity in AML often exhibit morphological relapse in a short period of time, so we hope to identify patients whose morphology has not yet relapsed but who are MRD-positive to avoid morphological recurrence. Esteve [15] studied 52 patients with acute promyelocytic leukemia (APL) and showed that when rescue treatment was given at molecular recurrence, prognosis was better than the treatment after reaching the current standard of recurrence.

Previously [16], we used IFN-α-1b and IL-2 combined with Tha to treat MRD-positive patients with AML. Results showed that the total effective rate of the ITI regimen in the treatment of MRD-positive AML was 72.2%. According to the MRD level before treatment, the effective rate of patients with MRD ≥ 1.0% was 57.1%, and the effective rate of patients with MRD < 1.0% was 81.8%. In this study, MRD in 20 patients was not stable in the negative state and had a high recurrence rate of economic or physical conditions, while some patients could not undergo allo-HSCT. Compared to other immunomodulators and targeted drugs, the prices of these three drugs were lower, and they were all within the scope of medical insurance reimbursement. Therefore, the ITI regimen was used to treat patients with AML1-ETO fusion gene–positive AML, and results showed that the total effective rate was 90%, which was higher than that of previously reported data, which might be related to the disease subtypes of the patients, which also suggests that the ITI treatment scheme is more effective in reversing AML1-ETO fusion gene positivity in patients with AML.

Taken together, these data suggest that the combination of interferon, interleukin-2, and thalidomide reduces the level of MRD in hematological remission, aiding patients AML1-ETO fusion gene–positive AML in reaching negative or decreased MRD, showing a dose–response relationship. A larger sample size is needed for further observation and follow-up to confirm these findings.
